# H-WORK Project: Multilevel Interventions to Promote Mental Health in SMEs and Public Workplaces

**DOI:** 10.3390/ijerph17218035

**Published:** 2020-10-31

**Authors:** Marco De Angelis, Davide Giusino, Karina Nielsen, Emmanuel Aboagye, Marit Christensen, Siw Tone Innstrand, Greta Mazzetti, Machteld van den Heuvel, Roy B.L. Sijbom, Vince Pelzer, Rita Chiesa, Luca Pietrantoni

**Affiliations:** 1 Department of Psychology, Alma Mater Studiorum—University of Bologna, 40126 Bologna, Italy; davide.giusino2@unibo.it (D.G.); rita.chiesa@unibo.it (R.C.); luca.pietrantoni@unibo.it (L.P.); 2 Institute of Work Psychology, Sheffield University Management School, University of Sheffield, Sheffield S10 FL, UK; k.m.nielsen@sheffield.ac.uk; 3 Institute of Environmental Medicine, Karolinska Institute, 171 65 Stockholm, Sweden; emmanuel.aboagye@ki.se; 4 Department of Psychology, Norwegian University of Science and Technology, N-7941 Trondheim, Norway; marit.christensen@ntnu.no (M.C.); siw.tone.innstrand@ntnu.no (S.T.I.); 5 Department of Education Studies, Alma Mater Studiorum—University of Bologna, 40126 Bologna, Italy; greta.mazzetti3@unibo.it; 6 Department of Work and Organizational Psychology, University of Amsterdam, 1018 WS Amsterdam, The Netherlands; m.vandenheuvel2@uva.nl (M.v.d.H.); r.b.l.sijbom@uva.nl (R.B.L.S.); v.pelzer@uva.nl (V.P.)

**Keywords:** mental health, small medium enterprises, public sector, multilevel analysis, Covid-19 pandemic

## Abstract

The paper describes the study design, research questions and methods of a large, international intervention project aimed at improving employee mental health and well-being in SMEs and public organisations. The study is innovative in multiple ways. First, it goes beyond the current debate on whether individual- or organisational-level interventions are most effective in improving employee health and well-being and tests the cumulative effects of multilevel interventions, that is, interventions addressing individual, group, leader and organisational levels. Second, it tailors its interventions to address the aftermaths of the Covid-19 pandemic and develop suitable multilevel interventions for dealing with new ways of working. Third, it uses realist evaluation to explore and identify the working ingredients of and the conditions required for each level of intervention, and their outcomes. Finally, an economic evaluation will assess both the cost-effectiveness analysis and the affordability of the interventions from the employer perspective. The study integrates the training transfer and the organisational process evaluation literature to develop toolkits helping end-users to promote mental health and well-being in the workplace.

## 1. Introduction

Mental health problems’ incidence, severity and burden has increased among the European population in recent years [1,2,3]. Common mental disorders such as psychological distress, anxiety and depression are the most frequent, especially among the active working population [4], whereby these are considered the main contributing factors to sickness absence [5,6,7]. In addition, the recent Covid-19 pandemic outbreak has triggered high levels of those symptoms in workers [8,9,10]. Although mental ill-health is an important cause of absence from work, it is also linked to high levels of presenteeism, whereby an employee remains at work despite symptoms leading to a reduced productivity [11]. The probability that workers in poor mental health report a decrease in productivity at work is about six times higher than for workers in good mental health [1]. As a result, the toll that mental health conditions takes on workers’ productivity translates into considerable economic costs for companies, employees and society at large, costing more than 4% GDP [12,13]. This adds to the inherently human and social costs related to poor mental health.

The European Agency for Safety and Health at Work [14] documented how common mental disorders, absenteeism, unemployment and long-term disability can be directly linked to work-related stress conditions that are growing strongly in several EU Member States. The understanding of predictors and outcomes of occupational well-being is well documented in the literature [15,16,17], and further knowledge is now also available for specific areas of interest such as digital interventions [18,19] and health promotion in small businesses [20]. Addressing work-related stress and adverse working conditions is particularly important to prevent prolonged stress conditions that lead to the incidence of serious mental health problems [21]. Therefore, a major challenge is to create mentally healthy workplaces. Consistently, in the field of work and organisational research, practitioners (e.g., industrial and organisational psychologists, human resource development practitioners, occupational health professionals) and researchers have acquired a broad and comprehensive knowledge of the psychosocial factors that can act as protective factors that enrich the workplace and support the creation of a healthy working environment. Indeed, high levels of psychological well-being are associated with improved work performance, lower turnover rates, qualitatively enhanced interpersonal relationships with colleagues and supervisors, higher levels of creativity and innovation, and higher organisational reputation [22].

An alarming fact is that although 79% of European managers say that they are concerned about stress and mental disorders in their workplaces, less than 30% of workplaces in Europe have procedures in place to deal with them effectively [14]. Due to a lack of knowledge and guidance, deciding which interventions should be implemented is a common issue facing employers. One of the main challenges is therefore to support organisations and their representatives (i.e., managers, supervisors, leaders) in recognising psychosocial risk factors as a critical issue that needs to be addressed by concrete measures. Nevertheless, managers and supervisors often lack training on mental health issues and can hardly rely on the support of specialists, as the link between work and health is not sufficiently established [23].

It is strategic to provide a deeper understanding as well as a greater awareness of what the necessary conditions for the implementation of effective interventions are to promote mental health and well-being at work. In recent years, increasing attention has been paid to the processes that may ensure successful implementation of interventions to promote workplace mental health and improve working conditions [24]. Providing employers with results highlighting not only interventions’ effectiveness, but also process mechanisms facilitating or hindering their impact, could make a relevant contribution when employers are faced with the challenge of managing and implementing changes in the work environment. In other words, it is increasingly crucial to establish the conditions that ensure that interventions are implemented according to plan and achieve their intended outcomes [25]. These conditions refer to contextual factors and processes such as management support and sponsorship, implementation strategies, and employees’ needs, preferences, perceptions and beliefs [26]. By enabling the development of integrated prevention strategies and the provision of effective methods and tools for monitoring mental health and levels psychological well-being in the workplace, it is possible to better understand the negative effects of work-related stress and to implement appropriate measures to counteract them.

In promoting mental health and well-being of workers and developing psychologically healthy workplaces, there are many types of initiatives that organisations can adopt. Workplace well-being interventions refer to formal or informal, planned, science-based, behavioural or psychological actions facilitating employee well-being by either increasing individuals’ resilience and coping resources or removing or modifying job stress causes [27]. Workplace well-being interventions should introduce changes, new work practices, programs, training, processes and policies at different levels of the organisation [28]. The scientific debate expresses concerns about the issue that the majority of studies on occupational well-being tend to adopt a relatively simplistic single-level model [29]. That is, when it comes to deepening our understanding of the psychosocial and organisational factors involved in preserving and sustaining a healthy workplace, literature studies these processes, and their effects, mainly at an individual employee level of analysis and intervention. On the contrary, there is a growing scientific awareness of the need to embrace multilevel approaches that may explain cross-level interactions and synergistic effects of workplace interventions [30,31].

The EU H2020 H-WORK project aims to promote mental health in public workplaces and small- and medium-sized enterprises (SMEs), as these types of workplaces constitute a particularly vulnerable target since often having little resources to manage workers’ mental health and psychosocial well-being [32,33]. In H-WORK, mental health promotion is conceived as “the process of enhancing protective factors that contribute to good mental health” [34]. In this sense, mental health promotion implies the development of individual, social and environmental conditions, which enable optimal health and promote personal empowerment and development. Mental health promotion initiatives involve the active participation of individuals in the process of achieving positive mental health and well-being and enhancing quality of life. It is an enabling process done by, with and for the people [35], which needs to be distinguished from mental illness/disorder preventions which, rather, aims to reduce the occurrence, frequency and re-occurrence of mental disorders or the risk of a mental illness, preventing or delaying their occurrence, and also decreasing their respective impact on the individuals, their families and society at large [35].

Particularly, the project employs a multilevel approach whereby interventions are developed and implemented at the individual, group, leader and organisational levels with the aim to achieve synergistic effects. The Consortium’s experts are in charge of evaluating the interventions carried out by gauging participants’ perception of the intervention activities implemented and their impact at all levels of the organisation (e.g., employees, work groups, leaders involved in training courses and middle/senior managers, representatives of the organisation) in order to understand the working mechanisms and outcomes of these multilevel interventions. The aim of the evaluation is to capture participants’ appraisals of the intervention activities, the extent to which they are integrated into daily work practices and procedures or are changing the way participants react cognitively or emotionally to the organisational reality. The project brings together seven academic partners, four private companies, one public institution and two European professional networks in order to design, implement and evaluate multilevel workplace mental health interventions in ten different organisations spanning across five European countries (i.e., Italy, Spain, Germany, the Netherlands, and Czech Republic).

Based on the recent results on cross-level interactions and effects between organisational, leader, team, and individual level, there is the need to develop advanced theory and empirical evidence to better support the adoption of multilevel approaches in the future [29]. Specifically, the project aims to develop methodological tools that should guide end-users (i.e., managers, practitioners and stakeholders) in: (i) assessing psychosocial risk factors in a particular work environment, (ii) deciding which interventions to implement, and (iii) evaluating both the process and the outcomes of the implemented actions in terms of increased workplace psychological well-being and cost-effectiveness. These instruments are the H-WORK Assessment Toolkit (HAT), the H-WORK Intervention Toolkit (HIT) and the H-WORK Evaluation Toolkit (HET), which are described hereinafter. As a main outcome, the project is expected to provide employers, managers and policymakers with both theoretical knowledge and practical toolkits for promoting workers’ mental health and psychological well-being from a multilevel perspective.

In line with the main objective of the project, the remainder of this concept paper is organised as follows. First, the main theoretical pillars of the project are presented. Second, the three research and intervention protocols feeding the H-WORK toolkit are described in detail. Finally, the results that the project is expected to achieve are discussed, along with some implications both for theory and practice aiming to create mentally healthy working environments.

## 2. Materials and Methods

### 2.1. Conceptual Model

The H-WORK project is based on four main theoretical pillars: (i) the integration of the Job Demands–Resources theory and principles from Positive Psychology, (ii) the IGLO model, i.e., multilevel interventions at the individual, group, leader and organisational levels, (iii) the adoption of a participatory approach, and (iv) the use of digital technologies.

#### 2.1.1. Job Demands–Resources Theory and Positive Psychology

The Job Demands–Resources (JD–R) theory [36] considers the work environment as a potential source of good mental health and well-being. According to this model, the work environment can be conceived as a constellation of job demands and resources, which exert an influence on workers’ health and psychological well-being. On the one hand, job demands refer to physical, psychological, social or organisational demanding aspects of the job that require physical or psychological effort from workers. On the other hand, job resources refer to physical, psychological, social or organisational aspects of the job that workers can use to counterbalance costs in terms of physical, cognitive and emotional energy. Job resources are also intrinsically motivating and may help employees to fulfil their basic needs, achieve work-related goals and positively influence personal growth and development. Although both job demands and job resources can independently impact individual well-being, job resources may buffer job demands by enabling employees to cope with job demands. Examples of job resources are autonomy and skill variety (i.e., individual structural resources), performance feedback and support from colleagues and supervisors (i.e., social resources), and role clarity, job control, pay, job security and career opportunities (i.e., organisational resources). Conversely, examples of job demands are workload, emotional demands and time pressure. The category of job demands has recently been differentiated into jobs that hinder the optimal functioning of the individual, and job challenges that, on the contrary, stimulate work engagement [37]. When job demands are prolonged and exceed job resources (i.e., a mismatch occurs between job demands and job resources), they can lead to strain, undesirable outcomes such as poor physical or mental health and well-being. Specifically, the JD–R theory posits the existence of two distinct processes leading to mental health, well-being and productivity of workers. First, the health impairment process whereby high job demands are causally linked to burnout over time. Second, the motivation process whereby high job resources result in increased work engagement.

Two main lessons from the JD–R theory especially inform the H-WORK project. First, workers’ mental health and well-being can be protected or enhanced by identifying and restoring the balance between job demands and resources characterising a specific work environment, which therefore needs to be managed and organised properly. Second, in order to preserve the balance, not only negative (i.e., demands) aspects of the job should be diminished, but also positive (i.e., resources) aspects should be promoted.

Consistent with this latter point, the Positive Psychology perspective assumes that people feel better when they manage to take a balanced view on positive and negative components of experiences, exercise gratitude and (re)frame events in a positive light and devote more attention to how to exploit their strengths rather than to ruminate on their weaknesses [38]. Additionally, Positive Psychology posits that benefits derive from mastering some personal resources (e.g., optimism, self-efficacy, hope, psychological capital), which recent studies have integrated into the JD–R theory [39]. More specific to the aims of the project, H-WORK follows the principles of Positive Occupational Health Psychology (POHP), which has been defined as: “the study and application of optimal functioning in the workplace. It promotes occupational health and flourishing, and examines how positive phenomena (contexts, personal resources) can be used to protect against occupational risks” [40].

JD–R and positive psychological interventions in the workplace have been shown to be beneficial for workers’ mental health [41,42], although conclusions and claims about their (in)effectiveness require more attention to contextual factors and other moderating and mediating variables [43]. To this end, the H-WORK project adopts a realist evaluation approach [44], which aims to identify mediators (i.e., working mechanisms) and contextual factors (i.e., moderators) that influence the interventions’ outcomes. In summary, the H-WORK project relies on the integration between JD–R theory and positive psychological principles in that, even if poor workplace mental health conditions should not be detected by the designed organisational needs assessment activities, interventions could still be implemented in order to enhance positive aspects of the work environment. This approach allows maintaining psychological well-being at satisfactory levels at each specific intervention site, thus preventing the emergence of workplace mental health issues.

#### 2.1.2. IGLO Model

As previously illustrated, job demands and job resources can be found at different levels. The IGLO model [23], from which the H-WORK project adopts its multilevel approach towards workplace mental health interventions, points to four different levels of analysis and subsequent interventions, namely the individual employee (I), the group or work team (G), the leader (L) and the organisation (O) levels.

Traditionally, there has been a debate whether interventions targeting the individual or interventions taking a preventive approach to change working conditions are more effective, and literature reviews have focussed on comparing the two [45]. This approach has been criticised for comparing apples and oranges [46] for several reasons. First, individual-level interventions target symptoms whereas organisation-level interventions target causes of poor health and well-being. Second, individual-level interventions target workers with identified problems whereas organisation-level interventions target the entire work population aiming at preventing poor well-being and mental ill-health. Third, individual-level interventions often focus on the immediate effects, namely, effects after three months [47], whereas systems change in organisation-level interventions take longer to implement and to take effect, with follow-ups up to three years [48]. An integrative literature review [49] concluded that multilevel interventions targeting both individuals and organisational practices and procedures were more effective than either individual-level or organisation-level interventions on their own. More recently, a meta-analysis [50] exploring whether resources at the individual, group, leader and organisational level differed in their prediction of well-being and performance, found no such differences and argued that interventions should target all four levels.

According to the IGLO model [23], sources of mental health and well-being at work exist at all levels of the organisation. Therefore, for interventions to reach optimal degrees of effectiveness, they should tackle all such levels or, where this is not feasible or justified, at least several of them. Building on this and on insights from previous literature [51,52,53,54], the H-WORK project will intervene by focusing on primary and secondary interventions at multiple levels of the involved intervention sites in order to also investigate the cumulative or synergistic effects deriving from the implementation of multilevel workplace mental health interventions. Primary interventions are aimed at preventing employees from developing health issues. As such, primary interventions focus on the elimination of stressors in the environment. Secondary interventions tend to focus on employees who may be at risk for developing mental health and well-being issues or employees in vulnerable subgroups. The objective for secondary interventions is thus to support individuals in dealing with stressors, thus boosting resilience [55].

#### 2.1.3. Participatory Approach

According to a bottom-up perspective on mental health promotion in the workplace, employees should not be seen as passive subjects but rather as active actors able to change their work environment [29]. Bottom-up, participatory research and intervention approaches have been supported by previous literature [26,46] because they (i) ensure the use of key stakeholders’ local knowledge of what the key issues are concerning job demands and resources, (ii) what changes need to be made and how, and (iii) ensures stakeholders feel valued, empowered and looked after. For instance, an intervention study [56] found that workers resisted interventions if they had not been involved in the participatory decision-making process of the intervention. By using a participatory approach, workers and their managers collectively gain resources, knowledge, and skills to identify workplace problems, develop solutions, and implement changes to improve their working conditions.

#### 2.1.4. Use of Digital Technologies

Digital-based interventions for workplace mental health can be defined as structured actions that aim to promote mental health at work by exploiting the potential offered by digital technologies. These may correspond to interventions originally designed to take place in physical presence and subsequently adapted to digital formats provided by online teleconferencing platforms. In addition, they can be interventions available through computer or smartphone apps only. Systematic literature reviews and meta-analytical evidence [18,19] have shown that digital technologies may be effective in improving workers’ mental health. Particularly, as far as physical distancing and avoidance of social gatherings will be among the best public health and safety measures to be adopted to counter the recent Covid-19 pandemic outbreak [57], and governments subsequently establish consistent rules worldwide [58], the importance of digital solutions for workplace mental health promotion will become apparent. In this context, workplace research and intervention activities will need to happen remotely, including those regarding mental health and psychosocial well-being at work. Therefore, digital-based interventions will be a feasible solution to deal with the negative psychological impact of the current pandemic, such as increased depression, anxiety, and stress among the working population, encompassing not only front-line health care workers as obviously the most at-risk occupational group [59], but also other workers providing essential services and customer services [8].

The H-WORK project will address the impact of Covid-19 on workplace mental health with specific actions oriented towards the implementation of tailor-made organisational interventions based on the potential offered by digital technologies, including a constant monitoring of the crucial mechanisms involved.

### 2.2. Study Design Overview

**Figure 1:**
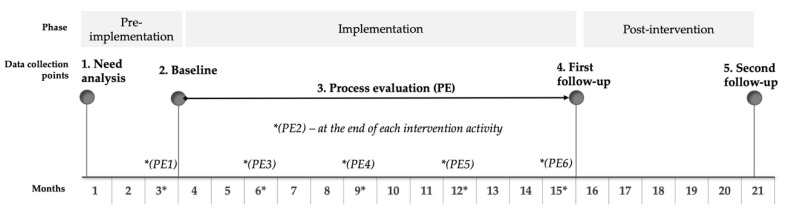
The H-WORK Study Design Overview.

Based on the theoretical framework presented above, the H-WORK study design comprises three major phases: a pre-intervention phase, an implementation-intervention phase, and a post-intervention phase, which are shown in Figure 1. Data will be collected at five main time points, including (1) the need analysis, (2) the baseline data collection (i.e., pre-intervention), (3) the process evaluation (i.e., implementation phase), (4) the first follow-up data collection and (5) the second follow-up data collection (i.e., post-intervention phase). First, the needs analysis (i.e., pre-intervention phase) is aimed to gather insights into which needs line managers and workers have to ensure their mental health and psychological well-being. Based on the results from the needs analysis, specific, tailored and multilevel interventions will be selected and implemented (i.e., implementation-intervention phase). Subsequently, the baseline data collection (i.e., pre-intervention phase), the first follow-up data collection and, six months later, the second follow-up data collection (i.e., post-intervention phase) will be performed to evaluate the effectiveness of the implemented interventions in terms of outcomes (i.e., increased workers’ mental health). Throughout the implementation phase, process evaluation will be carried out before, during and at the end of the intervention, to evaluate the implemented interventions in terms of both improved working mechanisms, mental health and well-being [60] and cost-effectiveness. More in detail (Figure 1), the process evaluation involves six measurement points: the first point at the baseline data collection point (i.e., pre-intervention phase, PE1), which captures the organisational context; the second point during the implementation phase (PE2), at the end of each activity, which collects how the participants perceived the intervention activity; the third, fourth and fifth point, every three months after the start of the implementation phase (PE3-PE4-PE5), to explore how the intervention activity is integrated into daily work practices and procedures or the extent to which the intervention activity is changing the way participants react cognitively or emotionally to the organisational reality; the sixth point, at the first follow-up data collection (PE6), is conducted exploring whether changes in mental health and well-being management can be observed at the IGLO level. Based on a mixed-method approach, while the measures are the same for all intervention sites at time points one (PE1) and six (PE6), the measures at time points two to five (PE2-PE5) are adapted to the intervention level.

As a basis for collecting data through the different time points, the H-WORK project adopt a mixed-method approach. That is, both qualitative (i.e., focus groups and interviews) and quantitative (i.e., questionnaires) methods will be applied to evaluate the interventions implemented. As well, both subjective (i.e., self-ratings, perceptions) and objective (i.e., organisational indicators, HR archival information) data will be collected. Examples of objective data include number of employees, number of males and females within the organisation, mental health policies and initiatives already in place, levels of absenteeism, number of paid sick leave days taken in the previous years, number of injuries, and the total healthcare costs. Examples of subjective data include job demands and job resources such as job control, job security, social support, and relationship with the job, and so forth. Each type of method has its own strengths and limitations. The aim of combining different methods is to take advantage of each other’s limitations, to triangulate perspectives on the targeted study issues and acquire a comprehensive picture of each issue here considered. Indeed, there is a growing interest in integrating qualitative and quantitative methods to address the complex events and scenarios, which characterise organisations nowadays [61].

#### 2.2.1. Variables of Study and Statistical Analysis

A key challenge when evaluating the effectiveness of multilevel interventions is how to best capture the true effects of interventions. Cleary and colleagues [54] suggested three types of study design that can be used depending on whether one is interested into assessing the combined impact of multilevel interventions only, rather than both the separate and the interactive effects of the interventions at different levels. The first type of design investigates the combined effects of multiple interventions on a given-level outcome (e.g., individual-level outcome), even if each of the implemented intervention is meant to target a specifically related outcome. That is, it focuses on the purely additive effects determined by a number of interventions implemented at different levels. The second type of design explores the separate effects of multiple interventions on a given-level outcome, whereby each of the implemented intervention is meant to target every same outcome. Finally, the third type of design aims to study the effects of intervening variables at different levels. In other words, while measuring the effect of multilevel interventions on the outcomes at the individual level (i.e., well-being, health-related outcomes), the study design aspires to evaluate both the effect of the different intervention components at different level of the organisation as well as the independent and synergistic effect of influences from different levels on processes.

**Figure 2:**
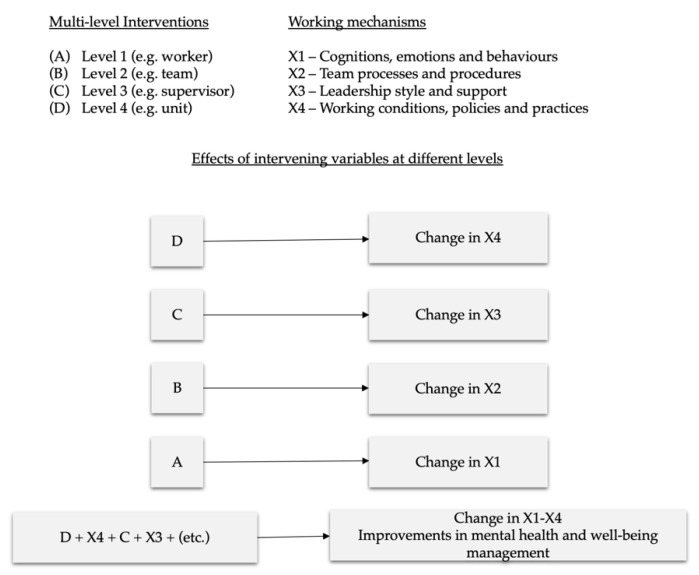
H-WORK design for assessing multilevel interventions.

As the H-WORK project aims to study not only interventions’ outcomes, but also processes and working mechanisms at different levels (i.e., individual, group, leader, organisation), it becomes essential to adopt a methodological framework in which the effects of multilevel interventions are observed by linking the outcomes at different levels (Change in X1–X4) to a process evaluation that entails each level of the organisation (D-X4; C-X3; B-X2; A-X1), as shown in Figure 2. In this sense, discovering the effects of a multilevel intervention programme should examine the chain of causality of effects approach [62]. In other words, it should be explored if, for example, the multilevel intervention has impacted the work environment through new working conditions which in turn, has led to changes in health, well-being and performance [62].

Any changes in these working mechanisms would lead together with improvements in the outcome of the multilevel interventions which is mental health and wellbeing. Indeed, as already highlighted by previous studies [26,62,63,64,65,66], more and more researchers are encouraging the use of statistical analysis capable of combining and linking process evaluation data with effect evaluation data in order to gauge the interactions of each of the intervention activity developed. In this sense, based on longitudinal approach, the use of multilevel regressions or (multi-group) structured equation models make it possible to include, for instance, knowledge on how participants reacted to intervention activities and contents, and, at the same time, process data closely related to the intervention attributes (e.g., participation, dropout) and to relate them to the expected outcomes (e.g., well-being). In this present research project, baseline levels act as context factors, process mechanisms as mediators linked to the outcomes (i.e., distal and proximal measures). Outcomes and process measures which will be adapted according to the type of interventions implemented as well as the levels of analysis involved (i.e., IGLO).

#### 2.2.2. Population and Sample Size

The H-WORK study sample will include 10 intervention sites, involving about 1500 participants, both employees and managers at all levels (i.e., senior management, middle management, line management), in five European countries (i.e., Italy, Spain, Germany, the Netherlands, and the Czech Republic), covering both public organisations and SMEs. Public workplaces from Italy, Spain, Germany and the Netherlands include two healthcare organisations, two higher education institutions, and one governmental organisation. Private sector workplaces from Spain and the Czech Republic include five SMEs from manufacturing, hospitality and ICT. Data will be provided by organisations (or representatives of organisations) including contextual information on the intervention site; leaders (managers or team-leaders within organisations); work groups (organisational functional areas or departments or work teams); and individuals (employees and employee representatives).

The sampling procedure for the implementation and the evaluation of interventions will be carried out through a quasi-experimental design that will include a waitlist control condition. Based on the presence of specific organisational constraints, participants will be randomly allocated to the intervention group or the control group. Sampling procedures will be carried out through the selection of a balanced proportion of males and females to guarantee equal opportunities of participation at the interventions and the subsequent evaluations. The project will also consider the actual gender distribution within a specific organisational setting (e.g., work team)

### 2.3. The H-WORK Toolkits

Carrying out the described H-WORK study phases will be possible through the deployment of the three research and intervention protocols that the project aims to develop, each one describing and providing a detailed framework as a basis for performing the planned activities. The H-WORK study consists of three components: Assessment (HAT), Implementation (HIT) and Evaluation (HET).

First, the HAT will provide guidance for the assessment of organisational needs in terms of workplace mental health. Second, the HIT will constitute a detailed toolkit of multilevel interventions, from which organisations will be able to choose based on the results from the need analysis. Finally, the HET will evaluate the process and outcome evaluation of the implemented interventions. Within the first H-WORK study component, the Assessment (HAT), a needs analysis will be conducted, including semi-structured interviews/focus group interviews with stakeholders and context measures, providing a better understanding of the specific needs regarding mental health and well-being in each organisation. In addition, baseline and follow-up quantitative data will be collected through an online survey to evaluate the effect of the intervention—including distal and proximal measures, after the interventions have been chosen. Within the second H-WORK study component, the Implementation (HIT), tailored interventions will be implemented in each intervention site. The intervention tailoring will be based on HAT’s needs analysis. The implementation phase ends with the final data collection at the end of the intervention period (i.e., first follow-up data collection). Within the third H-WORK study component, the Evaluation (HET), the effectiveness and cost-effectiveness of the implemented interventions will be evaluated. The evaluation is based on the process evaluation analysis and on the comparison of baseline and follow-up data collection points, with a second follow-up measure six months after the end of the interventions.

On the one hand, these three protocols will support the H-WORK partners in conducting research activities and implement tailored interventions while fostering methodological consistency across the different intervention sites involved. On the other hand, these toolkits will be conceived, designed and developed to enable effective decision-making and action-taking of relevant organisational stakeholders, such as managers, supervisors, leaders or policymakers, when it comes to identifying job demands and resources in the workplace and adopting measures or initiatives to counteract job demands and optimise resources. In this sense, the H-WORK toolkits are intended to be, at the same time, methodological instruments at the disposal of researchers, as well as practical guidelines for end-users.

#### 2.3.1. The H-WORK Assessment Toolkit (HAT)

The HAT consists of two parts, such as (i) a Needs Assessment Tool, and (ii) an Evaluation Tool. Consistent to the H-WORK conceptual model, the HAT activities will tackle multiple levels of the involved organisations (i.e., IGLO model), will leverage on both negative and positive aspects of work (i.e., JD–R theory and Positive Psychology), and will implement a bottom-up, participatory and mixed-method approach whereby employees, personnel representatives, middle managers and senior managers will take part in questionnaire-based surveys, focus groups, semi-structured interviews and stakeholder meetings.

Part 1 in the HAT, that is the Needs Assessment Tool, will provide guidance for the assessment of needs at multiple levels (i.e., IGLO) of the organisation in terms of workplace mental health. The aim is to inform an action plan and choice of interventions for improving mental health and well-being in the workplace. The needs assessment tool consists of (i) focus group interview, (ii) semi-structured interviews, (iii) contextual measure, and (iv) a plan for a stakeholder meeting and action plan.

First, focus group interviews will be conducted with employees and representatives (i.e., personnel representative, union representative, informal important employees in the working environment). The purpose is to gain mutual knowledge of psychosocial factors that affect mental health and well-being at work in general and to identify any actions and interventions needed to improve mental health and well-being in the workplace at individual and group level. In addition, it includes a specific section on how these identified issues discussed are impacted by COVID-19 and how the pandemic may influence their mental health and well-being.

Second, semi-structured individual interviews with middle and senior managers, which represent the “L” in the IGLO model will be conducted, the leader level. The aim is to understand the experiences, ideas and perspectives of the middle manager/senior manager around multilevel interventions to improve mental health and well-being in his or her employees. The rationale behind the interviews is to get the managers perspective and prioritizations and understand what they need in order to perform their role as manager successfully related to mental health and well-being issues [67]. In addition, the managers receive the same questions regarding the impact of COVID-19 as the employees in the focus group interview.

Third, the contextual measure is representing the “O” in the IGLO framework. It is designed to capture the extent to which management is committed to dealing with mental health issues, what sort of policies, practices and programmes are in place and how they are perceived. The survey covers three themes, such as (i) “Description of policies, programmes and practices”, to explore what measures and communication channels the organisation has for promoting mental health and well-being, types of benefits, compensations of facilitations available to workers and measures the organisation has for preventing stigma; (ii) “Perception of policies, programmes and practices,” consists of 8 items taken from the Workplace Integrated Safety and Health Assessment questionnaire (WISH-questionnaire) by [68]; and (iii) “Management commitment,” measured by a quantitative scale on psychological safety climate [69,70].

Finally, a summary of the needs analyses as described above will be presented at a stakeholder meeting, a participatory process including all stakeholders. The purpose of the stakeholder meeting is to prioritise which needs they would like to preserve and improve and then to develop and decide upon an action plan and choice of multilevel interventions for improving mental health and well-being in the workplace. In general, it has been argued that the success of implementation of interventions is the result of the alignment of a top-down commitment by the organisations’ authorities with bottom-up actions [71].

Part II of the HAT, the Evaluation Tool, includes proximal and distal measures which will be performed to evaluate the implemented interventions in terms of achievement of the desired outcomes. Proximal measures are quantitative, subjective, employee-centred measures that will indicate the direct and causally closest outcomes of each H-WORK intervention. As they are specific and intervention-dependent indicators of workplace mental health, they may be different across both interventions and organisation (i.e., intervention sites). Examples of proximal measures include work ability, work coping, mindfulness, strengths knowledge, strengths use, perceived stress, self-compassion, job crafting behaviours, and social support from co-workers and supervisors. In contrast, distal measures are quantitative, subjective, and employee-centred measures that will indicate the indirect and causally far outcomes of each H-WORK intervention. They will indicate improvements in global workplace mental health following the implementation of H-WORK interventions. As being general and comprehensive indicators of workplace mental health, they will be the same across both interventions and testbeds. Examples of distal measures include mental health and well-being (e.g., work-related anxiety, depression, stress, burnout, job satisfaction, work-family balance, mental health quality of life, work engagement) and organisational and performance-related outcomes (e.g., absenteeism, presenteeism, productivity loss, decreased work in-role and extra-role performance, turnover intentions, workplace mental health stigma).

In order to compare the situation before and after, distal and proximal measures are gathered again at the end of the multilevel interventions (i.e., first follow-up data collection, Figure 1) and six months after their conclusion (i.e., second follow-up data collection, Figure 1). In this regard, proximal measures are adapted to the different interventions carried out and the different organisational levels involved. On the contrary, distal measures are the same for the baseline and follow-up measurement points, thus ensuring the comparison of results.

#### 2.3.2. The H-WORK Intervention Toolkit (HIT)

The HIT aims to provide solutions that enhance workers’ mental health protective factors and reduce psychosocial risk factors through the adoption of multilevel interventions. Interventions will be tailored to the outcomes of the needs analysis and psychosocial risk assessment conducted as part of the HAT. The HIT Protocol will facilitate the implementation of multilevel interventions taking into account potential pitfalls while ensuring the effective participation of participants. The intervention phase represents the crucial and fundamental phase of the project as it ensures that each component of the intervention is measured and linked to the outcomes at different levels.

The HIT framework provides an overview of available interventions that can be used to either enhance resources or reduce demands at different levels in the organisation. Therefore, the HIT reflects the theoretical background used in H-WORK in terms of the JD–R approach as well as the IGLO model. The HIT consists of a toolkit in which information on interventions is contained in various ways. First, HIT includes 1) an IGLO-overview of interventions that can be effective in boosting mental health and well-being at each IGLO level; 2) a JD–R framework of interventions that target issues around job demands, job resources, as well as personal resources (e.g., lack of support, high role ambiguity, work-home conflict or lack of self-efficacy). This overview creates a link between interventions and relevant JD–R outcomes; 3) a multilevel JD–R based intervention-framework, which is a combination of the above (1 and 2) and should aid users in making effective decisions on which intervention to implement given the type of issues encountered at each level in the organisation. In order to be effective and easy-to-use, the HIT is presented with a clear set of guidelines on how to use the HIT (i.e., HIT Protocol) and preferably using a digital solution which can fully support the decision steps after an organisation has completed the HAT.

Criteria for selecting the interventions for the HIT include a solid theoretical base [72] and adaptability to fulfil the needs of various stakeholders in the organisation (i.e., employees, leaders, CEOs). In line with the focus of the H-WORK project to promote mental health, only primary and secondary interventions are included, as these are focussed on reducing stressors from the working environment and boost resilience and empowerment in dealing with stressors, respectively. The interventions in the HIT framework are organised and presented for each IGLO level. Examples of individual-level interventions encompass those based on mindfulness [41], job crafting [73], and training generally addressing personal resources [74] such as, for instance, work-anxiety coping strategies [75], employees’ self-efficacy [76], or workplace physical activity [77]. Group-level interventions typically address workshops on team cohesion, communication and performance [78]. Leader-level interventions may correspond, for example, to coaching-based leadership programs [79], increasing leaders’ capabilities to manage workers’ well-being and mental health, and leadership styles that may improve working conditions and workers’ mental health and well-being. Finally, organisation-level interventions might include, among others, job redesign activities, such as work shifts rescheduling [80,81] or changing working practices and procedures, and developing human resources and occupational health policies to support workers’ mental health and well-being.

For each intervention, the HIT framework will provide information in terms of: intervention theme (e.g., mindfulness-based), intervention name (as mentioned in the literature), purpose of intervention, intervention method (e.g., online training), timeframe, ease of implementation, the process working mechanisms, the content working mechanisms, the context requirements, the proximal and distal outcomes that can be customised and included into the HAT, the primary associated JD–R themes (e.g., communication), and references to articles that have tested or used the intervention. The main aim of the HIT is to help leaders, managers, policymakers to make an informed decision and select the most appropriate multilevel interventions based on the needs highlighted in the HAT analysis.

So, ultimately, the HIT framework functions as a database, where potential risks in the psychosocial work environment or vulnerabilities in employees, are linked to various types of interventions for each IGLO level. The database will be composed of interventions selected according to precise criteria and reflecting the theoretical basis of the project (e.g., focus on mental health and well-being outcomes; include theoretical mechanisms explaining why the content will work; have demonstrated a positive effect on mental health and well-being outcomes, using randomised-control trial, a quasi-experiment or at least pre- post measurement study designs; use validated outcome measures to examine effects on mental health and well-being; include information on the process of implementation). For example, if the outcome of the HAT is that emotional demands are an important issue among employees, then a stress management intervention can be recommended at the individual level and a group team building intervention can be recommended at the group level, because both interventions are likely to affect emotional demands. It might also be possible to target different needs from the HAT. For example, a stress management intervention at the individual level might be selected to minimise emotional demands, and a coaching-based leadership intervention to enhance supervisor support. In both cases, interventions are applied at multiple levels to improve the mental health of employees either directly or indirectly based on the specific needs identified. As such, the HIT framework will form an integrated whole rather than a collection of stand-alone tools. This is important, also given the current challenges with regards to how COVID-19 impacts workplaces, employees and their families. The HIT will therefore also include interventions suitable for tackling mental health issues as an outcome of the COVID-19 crisis.

#### 2.3.3. The H-WORK Evaluation Toolkit (HET)

The H-WORK Evaluation Toolkit (HET) aims to ensure continuous process evaluation of the HIT interventions along with the assessment of their economic impact. HET is closely related to HAT, as together they aim to explore both the conditions and mechanisms that lead to the desired results, in which circumstances interventions will be most effective, and how they can be transferred in the most effective way.

To reach this goal, the H-WORK project employs a realist evaluation approach [44]. In doing so, the project moves beyond answering the simple question of whether an intervention works to answering the questions of what works for whom under which circumstances [60]. Realist evaluation is used to overcome the dichotomy between process and effect evaluation, integrating both types of evaluation [81]. Central to realist evaluation is the development, testing and refinement of Context-Mechanism-Outcome (CMO) configurations [44]. These configurations are programme theories (to be developed according to the multilevel interventions chosen) aimed at testing under which circumstances (i.e., Context) the working ingredients (i.e., Mechanisms) of the intervention are triggered and bring about the intended effects (i.e., Outcomes). As the intervention content is developed, CMO-configurations are developed too, and realist evaluation focusses on testing these configurations. After testing the configurations, they may need to be refined, for instance, they may need to be altered based on the results of the performed evaluation [82].

**Figure 3:**
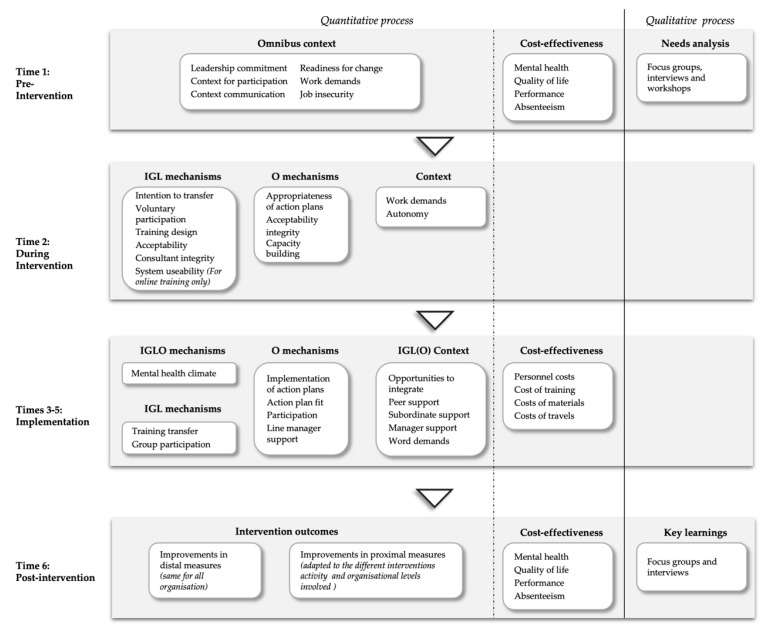
The H-WORK HET.

The H-WORK realist evaluation framework, emphasising the importance of ongoing evaluation to capture development in processes, is based on the framework proposed for the evaluation of organisational health interventions by Nielsen and Abildgaard [62], the Baldwin and Ford training transfer model [83] and the more recent Dynamic Transfer Model [84]. A key element of the HET is a mixed-methods approach to data collection, with multiple surveys at different phases of the H-WORK project supplemented with focus groups and interventions. In Figure 3, the overall evaluation framework is outlined.

Pre-intervention (i.e., Time 1), together with the baseline of the HAT, key contextual factors believed to influence whether mechanisms are triggered will be explored. Key contextual factors are management commitment to managing workers’ mental health and well-being [68,85] and the opportunities of workers to influence the strategies for managing mental health and well-being in the workplace. Equally important precursors of the intervention uptake is the communication about the intervention [68,86], as well as workers’ readiness for change [87], i.e., they see a need for change and feel the H-WORK project will enable the necessary change. Other important contextual factors that may influence uptake of the intervention is quantitative work demands and autonomy, meaning that workers need sufficient time and decision latitude to engage in intervention activities [88,89].

During the intervention (i.e., Time 2), data will be collected from the ongoing intervention activities themselves, be it training or workshops. For example, trainers and consultants need to be perceived to understand the needs of participants and the organisations, and the material and tools used as part of intervention activities need to support the process and learning, and there needs to be a supportive atmosphere where participants feel they can voice freely [90]. For online activities, the technological platform might be expected to be an important mechanism (i.e., usability) [91]. Voluntary participation is also important [92]. Intent to transfer may be an important intermediate outcome [84]. If workers do not intend to transfer skills and knowledge learned into changes in emotions, cognition and behaviours, intervention activities are unlikely to have a long-term effect. Where action plans have been developed, particularly at the organisational level, the participants’ appraisals of these action plans, i.e., that the action plans address the most pressing issues and will be impactful, will be measured [86].

Once intervention activities are implemented (i.e., Time 3-5), changes in cognitions, emotions and behaviours, including changes to work practices and procedures, need to be integrated into daily work and maintained over time in order to have a sustainable impact on mental health and well-being [83]. One important mechanism is overall changes in the attitude and ability to manage employee mental health and well-being, regardless of the level of intervention. Other significant process mechanisms include actual training transfer, that is the extent to which learned emotions, cognitions and behaviours are used in daily working life to minimise perceptions of demands and increase resources and for intervention including explicit action plans, these need to be implemented according to plan [89]. It is important that participants have the opportunity to influence the implementation process [26] and have the support of line managers and peers to make the necessary changes to emotions, cognitions and behaviours [84]. As in previous phases of intervention, it is important that participants have the opportunity to change their behaviours, they have opportunities to integrate changes to change emotions, cognitions and behaviours into their daily working life [84] and their workload is not so excessive that it prevents them from making changes [93]. By measuring the process mechanisms and contextual factors over multiple time points, we are able to capture the dynamic interactions between processes and context. For example, if a trainee attempts to change their behaviours, but the immediate manager and colleagues do not support such change, it is likely that we will see fewer attempts to transfer three months later [68]. We will feed back the results of the three-monthly surveys to participating organisations to enable adjustments to the implementation process. Such adjustments may increase the effectiveness of interventions as learning is implemented during the process, rather than extracted at the end of the project [94].

At the first follow-up (i.e., Time 6), information on the content mechanisms (i.e., proximal outcomes) and distal outcomes will be collected, including an overall measure of whether participants have experienced an improvement or deterioration of the ability to manage mental health and well-being in terms of their own ability as individuals, their work group’s ability, the ability of the leader and the organisation’s ability as a whole [95]. After the first follow-up, the comprehensive quantitative process evaluation will be supplemented with focus groups and semi-structured interviews with workers, managers and other key stakeholders to extract key learning about the intervention process [62].

A major limitation of existing workplace interventions is the lack of cost-effectiveness evaluation, in particularly in SMEs. To address this issue, the H-WORK project will perform an economic evaluation to assess the cost-effectiveness and business case (i.e., budget impact) of the implemented interventions from the employer perspective. For a more comprehensive description of the key characteristics (i.e., outcomes and cost measures) of the types of economic evaluation the H-WORK project will perform, see [96]. The baseline and follow-up data collection of distal measures will include outcomes (e.g., mental health, quality of life, performance) of each H-WORK intervention for the cost-effectiveness analysis. Concerning the cost data, when the employer’s perspective is applied, only health-related costs incurred by the employer are considered [97]. The employer expenses include both costs related to work absenteeism and presenteeism. Information on sickness absenteeism from work at baseline and follow-up will be obtained from participants to calculate costs related to absenteeism. In addition, self-reported presenteeism using the World Health Organization’s Health and Work Performance Questionnaire [98], will be obtained to evaluate the indirect workplace costs due to illness. For employers, an intervention’s effect on employee’s performance is highly important to the company’s bottom line. Presenteeism is particularly important because mental health issues are mostly chronic, which means presenteeism costs may exceed sickness absenteeism costs due to reduced work performance.

As part of the implementation phase, the cost of implementation component of each intervention at different levels are measured to evaluate the cost of these interventions. The costs of each H-WORK intervention will be collected using a bottom-up micro-costing approach (i.e., detailed data recorded on the number of resources consumed as well as their respective unit costs) [99], once every quarter after baseline until the first follow-up from employees to capture which resources were expended [100]. Intervention costs include all costs related to the implementation of intervention, which cover personnel costs (e.g., i.e., time needed for training, administration and implementation), and the costs of materials needed for implementation of the intervention (e.g., supplies or equipment including donated items). Healthcare utilisation costs included primary care costs, medication, and patient and informal care cost will not be collected because of the scope of the project capturing the costs for organisations rather than societal costs. However, individuals who participate in implementation-related activities may also experience indirect (i.e., “opportunity”) costs as a result of lost time spent on usual professional activities. Therefore, costs of travel and meeting time will be gathered only for participants, not on sickness absence to calculate the cost of lost time to participate in implementation-related activities. The mean cost at the different levels of implementation can be used to calculate a cost-effectiveness ratio or a net benefit (i.e., the mean differences between cost and benefits) of the interventions in monetary terms. The results of the cost-effective analysis will be presented as cost-effectiveness ratio (CER) and that of the net benefit will be presented as a cost impact to make a business case to employers.

The HET protocol has been designed to identify to whom and under what circumstances [60] the multilevel interventions have had an impact in terms of improved working conditions, mental health and well-being and at the same time evaluating their cost-effectiveness The post-intervention phase is aimed at collecting all the necessary data for evaluating the effectiveness of the interventions, their cost-effectiveness and to link the working mechanisms at each level of the organisation to the outcomes.

## 3. Discussion

Research conducted within the H-WORK project is expected to significantly contribute to the understanding of how to promote mental health at work, especially after the Covid-19 pandemic.

First of all, the H-WORK project will increase the knowledge currently available regarding the concept of “multilevel-ness” in the realm of workplace mental health interventions. The experiences that will be conducted in the ten European intervention sites, covering different sectors, both public and private, within a time frame of about three years, has in itself a suggestive potential that can expand the concept that we have of multilevel interventions as of now. As suggested by Bakker and Demerouti [29] the data available are scarce, and there is still much to be done to broaden our understanding of how the different levels (e.g., IGLO) can influence each other in a synergistic manner when it comes to promoting individual and organisational well-being.

In this sense, the use of a mixed-method approach allows to deepen the issue of multilevel mental health. In the iterative process designed for the H-WORK project, interviews, participatory focus groups together with standardised surveys and register data are collected before, during and after the implementation of multilevel interventions. This intervention strategy offers a relevant opportunity considering the knowledge acquired through data triangulation adopted in previous research and acquired from different organisational sectors [101,102,103]. The direct participation of managers, coordinators, employees, and safety representatives constitutes a pivotal point for the understanding of those factors that can facilitate or slow down a process of well-being improvement across multiple levels of the organisation. Staff inclusion as active agents of change, conceived as a necessary prerequisite for interventions within companies [104], can facilitate the understanding of the synergistic effects of multilevel interventions, an aspect that can significantly enhance the current design of existing improvement programs.

Realist evaluation plays a key role within this project, as it constitutes the primary means of monitoring the evolution of interventions implemented throughout the project itself. Within a longitudinal perspective, a constant monitoring approach at the four organisational levels (i.e., IGLO) enables the identification of contextual factors that can facilitate or hinder the implementation of interventions and their effectiveness. Considering the application of this monitoring strategy across different business and organisational contexts, the impact of the obtained results may be of great support for employers, stakeholders and policymakers in the implementation of new initiatives of multilevel interventions for mental health. The realist evaluation will highlight what worked, for whom and under what circumstances [60], deepen the knowledge of multilevel interventions prerequisite mostly for SMEs and public organisations which suffers from a lack of effective procedures and working mechanisms [14].

Overall, the H-WORK project adopts both a bottom-up and a top-down perspective on implementing workplace mental health interventions, complementarily. On the one hand, it is bottom-up because it considers employees’ participation and involvement, which may favour or obstruct intervention implementation and success depending on how it is managed. On the other hand, it is top-down because it considers the employer’s perspective in terms of financial-economic costs—which, again, may favour or obstruct intervention implementation depending on how cost-effectiveness awareness is managed. This would facilitate a shared understanding and more realistic expectations for the interventions, and hence a more successful implementation process.

Moreover, the substantial use of digital solutions for the promotion of well-being and mental health in the H-WORK project will bring new knowledge in this regard. Especially after the extraordinary event of the Covid-19 pandemic, this issue has taken an even more central role in the current landscape considering isolation, decreased physical activity, and increased rumination [105]. It is conceivable to imagine that organisations must tackle a great challenge in trying to preserve the health and safety of their employees and, especially in the public sector, of their customers. For this reason, an increasingly widespread adoption of digital solutions that are being tested and validated according to a scientific and evidence-based approach is desirable [106]. The contribution of the current project can lay the foundations for future digital solutions that will pursue the promotion of mental health according to a multilevel approach.

H-WORK will have an impact on the economic burden represented by the sum of increasing healthcare costs and decreasing labour productivity, reducing the overall costs that can be associated with poor mental health in the workplace, as well as improving workers’ mental health through properly designed and implemented health promotion interventions. Monitoring intervention sites’ performance, productivity and economic indicators throughout the project is an essential step in assessing the effectiveness of interventions in relation to their cost and impact on the organisational budget, although those issues are hardly addressed when it comes to implementing work strategies or interventions to promote well-being [107]. The affordability of interventions is another key point that will help and support senior management, decision-makers and policy-makers to highlight and understand the mechanisms that bring desired results in terms of mental health promotion at work, where these mechanisms are successful as well as cost-effective through informed decision-making practices.

The expected results of the project study should be considered in the light of the limits of the research. The present project study has embraced a multilevel analysis approach. However, an advanced version of the IGLO model [108] also includes the overarching and social context i.e., IGLOO, [23], that is the national legislation and social welfare policy. In the H-WORK project, the focus concern interventions within the organisation as depicted in [108] while societal interventions are beyond the scope of the study, namely the second O of the IGLOO model. Furthermore, the economic analysis covering the IGLO-levels (individual, group, leader and organisational) in the organisation takes the provider or employer perspective, which means that it will consider cost/benefits within the organisation. However, the economic analysis will include some costs e.g., country-specific social security contribution which are decided at the national level while the interview conducted during the process evaluation will allow to consider the overarching context level see also [23] that is the impact of different national contexts. Future studies addressing the impact multilevel interventions designed to promote mental health in the workplace at the over-arching level of analysis either from an economic and societal perspective are highly encouraged.

## 4. Conclusions

This paper describes the theoretical foundations, conceptual model, study design and methodological approaches of the EU H2020 H-WORK project, which aims to design, develop, implement, test and validate multilevel interventions for promoting mental health in public workplaces and small- and medium-sized enterprises (SMEs). By highlighting the working mechanisms involved in mental health promotion at different levels of the organisation, the research should provide innovative insights on how to improve working conditions around Europe.
